# Sonic Hedgehog Signaling in Organogenesis, Tumors, and Tumor Microenvironments

**DOI:** 10.3390/ijms21030758

**Published:** 2020-01-23

**Authors:** Kuo-Shyang Jeng, Chiung-Fang Chang, Shu-Sheng Lin

**Affiliations:** Department of Surgery and Medical Research, Far Eastern Memorial Hospital, New Taipei 220, Taiwan; cfchang.gina@gmail.com (C.-F.C.); ffcc840922@gmail.com (S.-S.L.)

**Keywords:** hedgehog, smoothened, cancer stem cells

## Abstract

During mammalian embryonic development, primary cilia transduce and regulate several signaling pathways. Among the various pathways, Sonic hedgehog (SHH) is one of the most significant. SHH signaling remains quiescent in adult mammalian tissues. However, in multiple adult tissues, it becomes active during differentiation, proliferation, and maintenance. Moreover, aberrant activation of SHH signaling occurs in cancers of the skin, brain, liver, gallbladder, pancreas, stomach, colon, breast, lung, prostate, and hematological malignancies. Recent studies have shown that the tumor microenvironment or stroma could affect tumor development and metastasis. One hypothesis has been proposed, claiming that the pancreatic epithelia secretes SHH that is essential in establishing and regulating the pancreatic tumor microenvironment in promoting cancer progression. The SHH signaling pathway is also activated in the cancer stem cells (CSC) of several neoplasms. The self-renewal of CSC is regulated by the SHH/Smoothened receptor (SMO)/Glioma-associated oncogene homolog I (GLI) signaling pathway. Combined use of SHH signaling inhibitors and chemotherapy/radiation therapy/immunotherapy is therefore key in targeting CSCs.

## 1. Introduction

During mammalian embryonic development, primary cilia with microtubule-based cellular organelles protrude from the surface of the cell [1]. Primary cilia defects cause “ciliopathies”, adversely affecting the development of brain, kidneys, eyes, liver, and other organs. Acting as cellular antenna, primary cilia transduce and regulate several signaling pathways such as Sonic hedgehog (SHH) and Wingless-activated (WNT) [1]. Among the various pathways, SHH is one of the most significant.

SHH signaling remains quiescent in adult mammalian tissues. However, in multiple adult tissues, it becomes active during differentiation, proliferation, and maintenance [2]. Moreover, aberrant activation of SHH signaling occurs in cancers of skin, brain, liver, gallbladder, pancreas, stomach, colon, breast, lung, prostate, and hematological malignancies [3].

Tumor microenvironment/stroma can affect tumor development and metastasis [4,5]. The tumor microenvironment/stroma includes endothelial cells, immune cells, adipocytes, and activated fibroblasts (the so-called “cancer-associated fibroblasts” (CAFs)) [6]. CAFs fuel cancer cells via secreting soluble factors to trigger metastasis and chemoresistance. These triggers include extracellular acidification, inflammation, activation of matrix metalloproteases, and decreased efficacy of chemotherapeutic drugs [7,8,9,10,11,12]. In addition, SHH produced by CAFs could regulate the microenvironment for cancer progression [13]. The details of tumor microenvironment in each organ are discussed in the following sections.

This article reviews the recent studies of the role of SHH in organogenesis, tumors, and tumor microenvironments.

## 2. Sonic Hedgehog Signaling Pathway Includes Canonical and Non-Canonical Pathways

The SHH signaling pathway could be categorized into canonical and non-canonical pathways. The canonical SHH signal transduction pathway consists of main components such as the Patched receptor (PTCH1, PTCH2), a 12-domain transmembrane receptor, the Smoothened receptor (SMO), a 7-domain transmembrane receptor coupled to G protein-coupled receptor (GPCR), and negative regulatory protein suppressor of fused homolog (SUFU) and the Glioma-associated oncogene homolog (GLI) family of transcription factors (GLI1, GLI2 and GLI3) [14]. Tumors produce ligands to activate the SHH pathway in an autocrine/juxtacrine manner. SHH ligands remove the inhibition of SMO by PTCH. SMO can activate GLI to regulate target gene expression and affect migration/invasion, cell cycle, tumor growth, and cancer stem cells. Moreover, paracrine Hedgehog (HH) signaling is important for epithelial cancers [15]. HH ligands secreted by tumor cells activate the signaling in the surrounding stroma, which provides a favorable microenvironment for tumor growth.

The non-canonical SHH signaling could be classified into three types, including (1) PTCH-mediated, (2) SMO-dependent/GLI-independent, and (3) SMO-independent GLI activation [16,17]. A simplified, broad, non-canonical SHH signal is defined as any related SHH signaling pathway component that differs from the usual canonical SHH signaling pattern [18]. For example, activation of SMO or GLI may occur via other signaling pathways such as Protein kinase A (PKA), Guanosine triphosphatase (GTPase), Phosphoinositide 3-kinase (P13K)/mammalian target of rapamycin (mTOR), or Rho, to drive target gene expression. The detailed mechanism of non-canonical SHH signal transduction still remains elusive, but probably acts as an alternative activation pathway when the canonical SHH signal transduction does not work functionally, or when transduction needs to “escape” the canonical SHH signaling pathway during cytotoxic or inflammatory stress [16].

## 3. SHH in Organogenesis, Vasculogenesis, and Angiogenesis

SHH signaling is essential for cell growth and tissue patterning. The pathway involves the development of neural tube, lung, skin, axial skeleton, gastrointestinal tract, pancreas, and other organs, as well as the regulation of tissue homeostasis and stem cell behavior [19]. During brain development, enhancer Shh brain enhancer 7 (SBE7) could not only initiate Shh expression but also induce Shh, which controls craniofacial morphogenesis and the etiology of holoprosencephaly [20].

SHH signaling induces endothelial cells and connective tissue support cells to release proangiogenic factors (Ang1, Ang2, and VEGF) [21,22]. However, some studies suggest that the response of SHH is limited to mesothelial and smooth muscle cells but not to endothelial cells [23]. Geng et al. [24] mentioned that SHH signaling affects vasculogenesis and angiogenesis. Others have found that the lung displays decreased vascularization in SHH-deficient mice [25]. Overexpression of SHH induces hypervascularization of the neuroectoderm during the development of mouse embryos [26]. Mutant zebrafish with deficient SHH signaling develop abnormal circulation and vascularization such as a single axial vessel with no arterial markers [27,28]. Though SHH is important in embryonic vessel formation, its role in tumor vasculature remains unclear.

## 4. SHH in Organogenesis of Forebrain and Cerebellum and in Medulloblastoma

### 4.1. SHH in Organogenesis of Forebrain and Cerebellum

During brain development, SHH signaling plays an essential role in two phases. The prechordal plate elicits early ShHH signaling to overlay the prechordal plate. Then, the triggered SHH signaling affects the late neuronal differentiation of the forebrain. The prechordal enhancer *Shh brain enhancer 7* (*SEB7*) regulates SHH signaling to affect the development and growth of the forebrain [20].

Primary cilia are essential for cerebellar differentiation, and the proliferation of neuronal granule precursors is SHH-dependent [29]. Additionally, SHH orchestrates the development and maturation of the cerebellum [30]. During embryogenesis, activation of SHH signaling occurs in the ventricular germinal zone (VZ) and regulates the proliferation of VZ-derived progenitors. Purkinje cells also secret SHH, and SHH can sustain the amplifications of the postnatal neurogenic niches (consisting of the external granular layer, the white matter, the excitatory granule cells, and inhibitory interneurons). During development, SHH signaling plays a role in Bergmann glial differentiation and facilitates the foliation of the cerebellum [30]. The mammalian GPCR 37-like 1 exists specifically in cerebellar Bergmann glia astrocytes and affects the proliferation and differentiation of postnatal cerebellar granules as well as Bergmann glia and Purkinje neuron maturation [29].

SHH signaling affects fissure formation. GLI2 activates SHH-induced granule cell progenitor proliferation and drives initial fissuring [29]. In the late prenatal or postnatal stages, SHH sustains the expansion of the external granular layer [30,31].

### 4.2. SHH in Medulloblastoma

Grausam et al. reported that medulloblastoma (MB) is the most common brain malignancy in pediatric patients, carrying a high mortality of up to 30% and high heterogeneity [32]. This disease arises from the cerebellum and is associated with early leptomeningeal metastasis, recurrence, and poor prognosis. According to their transcriptional profiles, MB can be classified into four subtypes: (1) WNT-MBs, (2) SHH-MBs, (3) Group C with abnormal transforming growth factor 1 beta (TGF1β) pathway, and (4) Group D with tandem duplication of a-synuclein-interacting protein [33,34,35,36,37,38]. SHH-MBs occur in both children and adults [39,40]. The transcriptional and genetic profiles are differently expressed in infant MBs and adult MBs. SHH-MBs are correlated with aberrations of the components of the SHH pathway (PTCH1, SUFU, GLI transcription factors and SMO) [30,41,42,43,44,45,46]. In particular, several SMO mutations involved in MB tumorigenesis have been found (L225R, N223D, S391N, D338N, D477G, D473H, G457S) [47]. Grausam et al. found that SHH pathway inhibitors may decrease both the proliferation and metastasis of tumors in a mouse MB model [32]. Inhibition HH signaling by sonidegib (LED225) and vismodegib (GDC-0449) have anti-tumor activity in SHH-driven MB [48]. Sonidegib led to better objective response rates than vismodegib against SHH-driven MB among five different clinical trials.

### 4.3. SHH in the Microenvironment of Medulloblastoma (MB)

SHH signaling activity alone is not sufficient for advanced development of medulloblastoma. Brain tumors consist of tumors, stem-like cells, and tumor-associated components (stroma) including vascular cells, immune cells, astrocytes, microglia, and extracellular martrix. Some epithelial cancer tumors can trigger SHH signaling to the stroma which enhances tumor growth [49]. The SHH subgroup of MB could significantly increase the gene expression of tumor-associated macrophages [50]. Tumor-associated macrophages are abundantly present in SHH MBs. Patients with decreased macrophage count usually have significantly worse prognosis [51].

## 5. SHH in Organogenesis, Tumors, and Tumor Microenvironments of the Liver

### 5.1. SHH in Organogenesis of Liver

During embryogenesis, the SHH pathway plays an essential role in hepatic specification of endodermic progenitors [52]. Conversely, there is no activation of SHH pathway in the adult mature hepatocyte [53], and the presence of SHH, PTCH, and GLI1 in the normal adult liver is minimal. Moreover, during liver repair, both myofibroblasts and progenitors can produce and respond to SHH ligands. In addition, Hippo/Yes-associated protein (YAP1) is a downstream effector of HH signaling pathway for liver regeneration [54].

### 5.2. SHH in Liver Injury and Hepatocarcinogenesis

SMO regulates adult liver repair by enhancing epithelial–mesenchymal transition [55]. From animal studies, chronic liver injury has been found to activate the SHH pathway. After Fas-induced liver injury, SMO is upregulated in hepatocytes. HBx (HBV gene product HBx protein) transformation induces the activation of the SHH signaling pathway. Cai et al. found that the SHH signaling pathway is activated during hepatocarcinogenesis [52]. Activated SHH signaling facilitates the cell proliferation after enhancing the G2/M transition via an increase in cyclin B1 and cyclin-dependent kinase 1 (CDK1) [52]. SMO plays an essential role during early hepatocarcinogenesis [55]. Overexpression of SMO-mediated c-Myc affects hepatocarcinogenesis significantly [56]. In hepatocellular carcinoma (HCC) patients, SMO mutation at the C-terminal lysine (K575M) involves the binding between PTCH and SMO to alleviate SMO from PTCH suppression to activate the downstream signaling [57]. Liu et al. found that hypoxia-inducing oxidative stress, epithelial–mesenchymal transition (EMT), and activation of the non-canonical SHH signaling pathway aggravates the invasiveness of HCC cells [58]. Chen et al. found that the SHH pathway induces the migration and the invasion of HCC cells via activation of focal adhesion kinase (FAK)/P13K/AKT signaling-mediated matrix metalloproteinase-2, as well as matrix metalloproteinase-9 production [59]. Other authors found that the SMO inhibitor GDC-0499 could inhibit hepatocarcinogenesis in HBx transgenic mice [60].

The expression of SMO affects the prognosis of HCC patients [61]. The overexpression of SMO and an increased ratio of *SMO* mRNA to *PTCH* mRNA correlates with HCC size in patients [62]. Jeng et al. reported that the high expression of SHH signaling pathway molecules affects the risk of post-resection recurrence with HCC [62]. Wang et al. found that *SMO* polymorphisms in transplant recipients are associated with an increased risk of postoperative HCC recurrence [63].

### 5.3. SHH in the Microenvironment of Primary Liver Cancer

The most common primary liver cancers are hepatocellular carcinoma (HCC) and cholangiocarcinoma. HCC originates from hepatocytes and cholangiocarcinoma originates from bile duct cells.

The disruption or change of the liver microenvironment and immune cell composition mainly promotes the malignant transformation and progression of HCC [64]. When the liver regeneration microenvironment deteriorates, inflammation and vascular changes can occur to enhance hepatocarcinogenesis [65]. To improve the microenvironment in liver regeneration through a regulation of multi-component, multi-target, multi-level, multi-channel, and multi-timed factors, an updated strategy for liver cancer prevention or inhibition is required. Hedgehog signaling could promote tumor-associated macrophages (TAMs) and thereby lead to immunosuppression [66]. SMO expression in myeloid is required not only for HCC growth but also for M2 polarization of TAMs.

Cholangiocarcinoma remains the second most common primary malignancy of the liver. Razumilava et al. found that cholangiocarcinoma cells could express non-canonical SHH signaling with chemotaxis even when cilia function is impaired. The non-canonical SHH signaling pathway contributes to the progression of cholangiocarcinoma [67]. Fingas et al. described the use of cyclopamine (SMO inhibitor) as able to increase the apoptosis of cholangiocarcinoma cells. Cyclopamine also inhibited tumor growth and metastasis in a rodent model study [68]. El et al. noted that SHH signaling pathway inhibitors can enhance the necrosis of cholangiocarcinoma cell [69]. Moreover, myofibroblast-derived platelet-derived growth factor (PDGF)-BB-mediated cyto-protection in cholangiocarcinoma is dependent on the HH signaling pathway [68]. PDGF-BB could induce translocations of SMO to the plasma membrane. Therefore, SMO inhibitor could promote the apoptosis of cholangiocarcinoma cells as well as their metastasis.

## 6. SHH in Gallbladder Organogenesis, Gallbladder Cancer, and Tumor Microenvironment

### 6.1. SHH in Gallbladder Organogenesis

The genetic and in vitro studies found that the SHH signaling pathway is essential for the proper formation of smooth muscles downstream of *Sox17* in the development of the gallbladder during the late organogenesis periods [70].

### 6.2. SHH in Gallbladder Cancer

Matsushita et al. found a higher expression of SHH in human gallbladder cancer specimens compared to normal gallbladder tissue [71]. SMO inhibitors could inhibit the proliferation of cancer cells. Inhibition of gallbladder cancer cell invasiveness functions via the suppression of matrix metalloproteinase-2 (MMP-2) and MMP-9 and epithelial-mesenchymal transition [71]. SMO si-RNA-transfected gallbladder cancer cells underwent a decrease in tumor volume according to a xenograft study [71]. The expressions of SHH, PTCH, and GLI1 are upregulated in gallbladder cancer. Aberrant activation of SHH signaling protein could be found in chronic cholecystitis and gallbladder cancer. SHH expression increased in severe chronic cholecystitis but decreased after the progression to gallbladder cancer. High GLI1 expression is correlated with worse prognosis of gallbladder cancer [72]. Moreover, high expression of SHH-signaling molecules SHH, PTCH, and GLI are associated with poor survival in the gallbladder [73]. Several mutations of the *SHH* gene in gallbladder carcinoma could be identified and associated with the carcinogenesis [74].

### 6.3. SHH in the Microenvironment of Gallbladder Cancer

Patients with high levels of SHH-signaling molecules were found to be associated with unfavorable survival outcomes. It could be associated with inflammation states [75]. Inflammatory responses could drive cancer progression such as EMT, angiogenesis, and metastasis. However, the evidence for SHH in the microenvironment of gallbladder cancer is still required for the investigation.

## 7. SHH in Organogenesis of the Pancreas, Pancreatic Cancer, and Pancreatic Cancer Microenvironment and Cancer Stem Cells

### 7.1. SHH in Pancreas Organogenesis

During the development of the human pancreas, SHH signaling remains low in pancreatic progenitor cells. Between embryonic week 14 and 18, both SMO and GLI2 gradually start to accumulate in primary cilia [76,77]. Then, GLI3 becomes gradually lower in the nucleus and cytoplasm of ductal epithelial cells during pancreas development. SHH signaling is necessary for both proliferation and maturation of the pancreas [76,77].

### 7.2. SHH in Pancreatic Cancer

The primary cilium and receptor SMO have been demonstrated in the vessels and stromal fibroblasts of tumors, providing evidence of SHH signal pathway activation. One hypothesis has been proposed that states that the behavior of mesenchymal and endothelial cells is affected by SHH, which is secreted from pancreatic cancer in a paracrine manner [78,79]. Aberrant SHH expression occurs in the early stages and during the progression of pancreatic cancer. Expression then increases from pre-malignant to malignant lesions of the pancreas [78]. Kumar et al. also emphasized the essential role of SHH signaling in the development and metastasis of pancreatic cancer cells [80]. Niyaz et al. mentioned that dysregulated SMO could be a therapy target in pancreatic cancers [81].

Conversely, Tian et al. found that SMO expression in epithelial cells has no role in affecting the development of pancreatic cancer [82].

### 7.3. SHH in the Microenvironment of Pancreatic Cancer

Li et al. emphasized that the activity of the SHH pathway is low in normal pancreatic tissue, whereas in pancreatic adenocarcinoma, the activity of the SHH pathway signaling in tumor epithelia and surrounding stromal tissue becomes higher [79]. Saini et al. found that SHH is upregulated in both the stroma and epithelial compartments in poorly differentiated pancreatic ductal carcinoma [83]. Wang et al. suggested that tumor necrosis factor alpha and interleukin-1 beta in stromal hyperplasia could promote the growth of pancreatic ductal adenocarcinoma after the SHH pathway activation for both canonical and non-canonical models [84]. Bailey et al. supported the hypothesis that the pancreatic epithelia secretes SHH, which is essential in establishing and regulating the pancreatic tumor microenvironment to affect cancer progression [78]. A high expression of SHH enhances the size and metastasis of primary pancreatic tumors [78]. SHH ligands exhibited by pancreatic cancers promote tumor growth indirectly via SHH signaling activation in the surrounding stroma. This paracrine activation of SHH signaling in the tumor microenvironment affords an environment favorable for the proliferation, metastasis, and drug resistance of cancer cells [79]. Mouse models (subcutaneous and orthotopic implantation) using a pancreatic tumor cell line showed that cells can secrete SHH, and that expression of SHH was high in a transformed primary cell line. SHH significantly affects both microenvironment and tumor progression, and is a potential target to suppress the desmoplastic and metastatic processes involved in pancreatic cancer [78]. Rucki et al. suggested that dual inhibition of the SHH and hepatocyte growth factor (HGF) pathways in the stroma could significantly suppress cancer cell growth and metastasis [85].

The activated SHH in pancreatic tumors enhances angiogenesis, lymphangiogenesis, and metastasis to produce a pro-angiogenic effect and to promote metastasis in the stroma [78]. The effects on lymphangiogenesis on SHH are important and could affect the metastasis of pancreatic tumor cells to the lymph nodes [86]. Targeting the stroma of pancreatic cancer could improve drug delivery and inhibit both angiogenesis and lymphangiogenesis. Pitarresi et al. [87] proposed a mechanism of stromal fibroblasts enhancing pancreatic tumor cell growth. Tumors secrete SHH, which activates the SHH pathway in pancreatic fibroblasts [88]. The fibroblasts in the tumor microenvironment then promote tumor growth via the disruption of paracrine SHH signaling. Some investigators found that SHH antagonists may successfully suppress tumor growth in xenograft tumors [88], whereas Pitarresi et al. knocked out SMO in fibroblasts to enhance tumor growth. Pitarres et al. found that the SMO gene in stromal fibroblasts affected the proliferation of pancreatic cancer cells [87]. In turn, deletion of SMO could activate oncogenic protein kinase B in the fibroblasts [87].

However, Tian et al. found that only the tumor stroma is competent in transducing the SHH signal, given that SMO is activated in the mesenchyme [82]. These researchers used a mouse model of pancreatic cancer and found that SHH signaling activation is present in the SHH-expressing tumor epithelium surrounding the stroma. Using quantitative RT-PCR to examine tissue samples of both primary or metastatic human pancreatic cancer, activation of the SHH pathway in the tumor stroma was found. Researchers have suggested that SHH-mediated tumorigenesis is a paracrine model. Pancreatic tumor cells could secrete SHH ligand to induce the SHH target genes in the adjacent stroma, thus promoting tumor growth [82].

Rhim et al. found that SHH-deficient tumors with reduced stromal content became more aggressive. These tumors presented undifferentiated histology, increased vascularity, and heightened proliferation. The administration of vascular endothelial growth factor receptor (VEGFR) blocking antibody could improve the survival of SHH-deficient tumors, meaning that SHH-driven stroma inhibits tumor growth partly via restraining tumor angiogenesis [89]. Pitarresi et al. analyzed fibroblasts in a sample of patients with pancreatic cancer, demonstrating heterogeneous patterns of expression in the components of the stromal fibroblast [87]. They also found that patients with decreased stromal phosphatase and tensin homologs usually led to a worse prognosis. These data established the potential to modulate pancreatic cancer stroma for targeted therapy [87].

After SMO deletion, fibroblasts overexpressed *transforming growth factor-alpha* (*TGF-α*) mRNA, and TGF-α protein, resulting in activation of epidermal growth factor receptor signaling in acinar cells and in acinar-ductal metaplasia [90]. This means that a non-cell-autonomous mechanism could modulate Kras G12D-driven acinar-ductal metaplasia. Such a phenomena could be balanced through cross-talk between the SHH/SMO pathway and alpha serine/threonine-protein kinase/GLI2 pathways in the stromal fibroblasts [90]. Zhou et al. demonstrated that using SMO-positive pancreatic cancer cells, GDC-0449 could downregulate SHH signaling genes and reverse fibroblast-induced resistance to doxorubicin [91]. Liu et al. found that genetic ablation of SMO in stromal fibrosis could disrupt the paracrine SHH signaling with acinar-ductal metaplasia under a Kras G12D mouse model [90]. Kumar et al. designed a novel GDC-0449 analog 2-chloro-N ^1^-[4-chloro-3-(2-pyridinyl)phenyl]-N ^4^,N ^4^-bis(2-pyridinylmethyl)-1,4-benzenedicarboxamide (MDB5) to inhibit pancreatic cancer [80]. Using a mouse model, Olive et al. reported that SMO inhibitor enhances the vasculature within the tumor and facilitates the delivery of chemotherapy agents to pancreatic cancer [92]. Von Ahrens et al. emphasized that targeting SHH to act on the stroma and exploit the secretory capability of CAFs could enhance drug delivery and prevent chemoresistance in cancer cells [93].

### 7.4. SHH in Cancer Stem Cells of Pancreatic Cancer

SMO could affect epithelial–mesenchymal transition, invasion, and migration of cancer stem cells in the pancreas [94]. Wang et al. knocked down SMO to inhibit pancreas cancer stem cells that possessed characteristics of self-renewal, epithelial-mesenchymal transition, invasion, migration, lung metastasis, chemoresistance to gemcitabine, and tumorigenesis [94]. The inhibition of SHH signaling pathway by sulforaphane could alter the expression of stem cell-related genes such as *Nanog* and *Oct-4* [95]. Therefore, targeting cancer stem cells by SHH pathway could improve the outcomes of pancreatic cancer patients.

## 8. SHH in Organogenesis of the Gastrointestinal (GI) System, GI Cancer, the Microenvironment, and Stem Cells of GI Cancer

### 8.1. SHH in Organogenesis of the Stomach

SHH signaling affects foregut development [96]. Among the three SHH ligands in the mammalian genome, SHH levels are highest in the mucosa of the embryonic foregut [96]. Ranakho–Santos et al. emphasized that the SHH signal plays an important role in organogenesis of the mammalian gastrointestinal tract [97]. SHH plays a significant role during epithelial development and differentiation, homeostasis, and neoplastic transformation of the stomach [19,96,98]. Van den Brink et al. reported that in humans, there are abundant *SHH* mRNA and SHH proteins in the gastric fundus, but no SHH protein is present in the esophagus or intestines [97,98]. SHH is needed during the growth and differentiation of the esophagus [99]. High expression of SHH in parietal cells contributes to gastric acid production [96]. Myofibroblasts are the predominant cell to respond to SHH ligand in normal stomach tissue. SHH induces the epithelial phenotype in gastric organogenesis. SHH null mice show an overgrowth of gastric epithelium as patterned into glandular and nonglandular regions [100].

### 8.2. SHH in GI Cancer and its Microenvironment

#### 8.2.1. Stomach: Gastritis, Gastric Ulcer, Gastric Carcinogenesis, and Gastric Cancer Stem Cells

Ranakho–Santos et al. suggested that mutations to the SHH signaling pathway affect human gastrointestinal function [100]. Chronic inflammation caused by *Helicobacter pylori* (HP) infection causes parietal cell atrophy and metaplastic cell proliferation (a precursor to human gastric cancer) [96]. In a mouse study following HP infection, canonical SHH signaling-induced inflammatory cells were recruited from the bone marrow to the stomach along with metaplasia [96]. Gastric parietal cells secreting SHH affect the regeneration of the epithelium after gastritis following HP infection [101]. Dysregulation of the SHH signaling pathway causes the disruption of gastric differentiation, loss of gastric acid secretion, and the development of cancer [101]. Merchant et al. showed that overexpression of SHH in parietal cells induces gastric acid production. In an uninfected stomach, myofibroblasts are the predominant cells that respond to SHH ligand. Xiao et al. used a mouse model to find that ulcer healing occurs with decreased ulcer size, angiogenesis, macrophage infiltration, and granulation tissue formation upon re-expression of SHH within ulcerated tissue [102]. Re-expression of SHH affects gastric regeneration as well. In a mouse model following HP infection, canonical SHH signaling induces bone marrow to recruit inflammatory cells to the stomach, leading to metaplastic development. The transcription factor GLI1 regulates the polarization of invading myeloid cells and myeloid-derived suppressor cells to afford a microenvironment that favors wound healing and neoplastic transformation. In mice, GLI1 mediates a shift in phenotype to gastric myeloid-derived suppressor cells via inducing Schlafen 4 (slfn4) directly. These could be taken as biomarkers to predict gastric cancer progression and determine benefit after SHH antagonist treatments [96].

Atrophic change with loss of parietal cells also causes loss of SHH expression, indicating an early sign in the mucosa before cellular transformation [103]. The change of SHH expression induces gastric cancer development. SHH is primarily located in parietal cells of the gastric body. However, the intestinal type of gastric cancer mainly develops in the antrum. The method by which fundic SHH regulates proliferation in the antrum remains elusive. SHH regulates downstream targets, including PTCH and the TGF-beta family members (bone morphogenic proteins (BMPs)) [97]. The latter targets of the SHH pathway are present in the mesenchyme rather than the epithelium, suggesting that SHH regulates epithelial–mesenchymal crosstalk. It is likely that these mesenchymal factors are present preferentially in the antrum, whereas gastric atrophy and subsequent loss of SHH could remove the inhibitory signal that suppresses antral proliferation. Loss of SHH in the mucosa during HP-associated atrophic gastritis becomes an early change prior to cellular transformation. SHH plays an important role in sustaining gastric epithelial differentiation, and the loss of SHH favors early carcinogenesis. Yang et al. reported that a high expression of SMO and GLI1 is correlated with gastric carcinogenesis [104]. Other researchers found that SMO or GLI1 inhibitors impair the migration and invasion of gastric cancer cells [104]. Chong et al. mentioned that in gastric cancer cells, galectin-1 promotes cancer invasion and epithelial–mesenchymal transition via activation of the non-canonical SHH pathway [105]. Wu et al. found that GDC-0499 inhibits the proliferation of gastric cancer cell line SGC-7901 and accelerates apoptosis [106].

Surface markers of gastric cancer stem cells CD133 and CD44 were found to be significantly decreased in the SGC-7901 gastric cancer cell line following GDC-0499 treatment [106]. This SMO antagonist could affect the maintenance and other properties of gastric cancer stem cells [106]. In paclitaxel-treated gastric cancer cells, overexpression of SMO could reduce activated caspase 3, thus decreasing cancer cell death [107]. Ma et al. found that in paclitaxel-resistant gastric cancer cell lines, there was an overexpression of SMO. SMO overexpression upregulates 5-Bromo-2′-Deoxyuridine (BrdU) in gastric cancer cells [107].

#### 8.2.2. Colon Cancer

Zhang et al. found that when compared with normal colon tissue, overexpression of SMO and GLI protein is noted in colon cancer tissue and colonic adenoma tissue [108]. Li et al. reported that in colorectal cancer, SMO expression corresponds with tumor status and patient prognosis [109]. Ding et al. found that SMO expression is an independent biomarker for postoperative liver metastasis. Similarly, SMO plays an important role in colon cancer progression [110].

Colon cancer driven by cancer stem cells forms a heterogeneous tumor, and whole-transcriptome analysis has revealed enhancement of WNT and Hedgehog signaling in cancer stem cells. Canonical GLI-dependent SHH signaling negatively affects WNT signaling in intestinal tumors. Regan et al. found that the SHH signaling in colon cancer stem cells includes SHH-dependent, non-canonical PTCH1-dependent, and GLI-independent pathways, suggesting that non-canonical SHH signaling positively affects WNT signaling and is essential for the survival of colon cancer stem cells [111]. Niyaz et al. suggested that dysregulated SMO could be as a treatment target of colon cancer [81].

Wu et al. found that GDC-0449 inhibits the replication of colon cancer cells and triggers apoptosis via downregulating B-cell lymphoma 2 (Bcl-2) [112]. Magistri et al. found that GDC-0499 could suppress and modulate cellular plasticity and invasiveness of colorectal cancer [113].

## 9. The Role of the SHH/SMO Pathway in Breast Organogenesis, Breast Cancer, and Its Microenvironment

### 9.1. SHH in Organogenesis of the Breast

#### SHH Signaling During Normal Mammary Gland Development

Riobo-Del Galdo et al. emphasized that SHH signaling is essential in breast development and homeostasis. The expression of SHH component pathways in mammary tissue differ at different stages of development [114,115,116,117,118,119]. During embryonic development, the canonical SHH signaling pathway is inhibited in breast tissues [4,111], and SHH gene expression is affected temporally and spatially via genetic and epigenetic mechanisms [4,111]. In a mouse study, early mammary bud formation was found to require active repression of GLI1 by GLI3R [4]. During puberty, ductal morphogenesis is affected by canonical and non-canonical SHH signaling, for which type I non-canonical SHH signaling plays an essential role [4,111]. During puberty, the elongation of the terminal buds is affected via activation of cellular Src kinase (c-Src), estrogen receptor alpha (ERα), and extracellular signal-regulated kinase (ERK) cascades in mammary luminal epithelial cells [120,121,122]. At this stage, a decrease of the expression of SHH ligands GLI1, GLI2, GLI3, and PTCH1 in the mature mammary gland is also found [114,118,123]. In normal adult mammary tissue, this pathway becomes downregulated.

### 9.2. SHH in Breast Cancer

In transgenic mice, active SMO with high canonical signaling activity may be involved in the development to mammary ductal dysplasia [4,124,125].

SHH contributes to tumorigenesis and progression with some types of breast cancer [111]. SMO expression is present in ductal carcinoma in situ (DCIS) and invasive breast cancer (IBC), but is absent in normal breast tissue. SMO expression affects tumor size, lymph node involvement, and tumor recurrence. However, it does not affect histological grade or other oncology markers [111]. SMO expression does not correlate with PTCH1 expression in either DCIS or IBC. This means that the activation of the SHH pathway cannot regulate SMO. Such evidence suggests that targeting downstream molecules of SMO when treating breast cancer may not be effective [111].

Guerrini et al. found that the SHH signaling pathway regulates breast cancer cell migration and invasion through carbonic anhydrase (CA) Ⅱ [126]. SHH pathway activation affects breast cancer metastasis [127]. Many studies support the claim that target genes *GLI1* and *GLI2* are involved in breast cancer cell proliferation, survival, migration, invasion, EMT, angiogenesis, and osteolytic metastasis [4,128,129,130,131,132,133,134,135]. Benvenuto et al. used an SMO inhibitor (GDC-0449) and GLI inhibitor (GANT-61) to target the SHH/GLI pathway to inhibit breast cancer cell growth in both in vitro and in vivo studies [136]. The researchers found that in breast cancer, downstream SMO targeting is better than upstream SMO when attempting to interrupt SHH signaling [136]. The development of highly vascularized tumors is regulated by overexpression of SHH, which affects the pro-angiogenic transcription factor cysteine-rich angiogenic induced 61 (CYR61) in a GLI-dependent manner [4,131]. Han et al. found cancer stem cells to be heavily present in breast cancer via non-canonical SMO-independent SHH signaling activation [137]. SHH inhibitors are therefore another therapeutic option [138].

### 9.3. SHH in Estrogen Receptor-Positive Breast Cancer

Recently, studies of estrogen receptor (ER)-positive breast cancer (BC) cell lines have revealed that estrogen can increase GLI1 and GLI2 [4,139]. However, GANT61, which inhibits GLI1 and GLI2 activity, could reduce the proliferation of cancer stem cells in culture. GLI transcription factors as mediators were also able to affect estrogen in BC [139]. Some authors found that estrogen affects overexpression of SHH and GLI1, activating SHH signaling and enhancing invasiveness of the ER-positive T47D (HER2-) and BT-474 (HER2+) cells [140]. These results suggest that cross-talk between ER- and SHH-signaling pathways facilitate the invasiveness of ER-positive BC cells [4]. The association between GLI1 and ER (luminal subtype marker) remains elusive [4]. In ER-positive breast cancer, overexpression of GLI1 affects early disease onset, higher SHH expression, higher Ki-67 index, higher histological grade, advanced stage, lymph node metastasis, and both shorter disease-free survival and overall survival. Overexpression of GLI1 acts as a predictor in age, ER-positive expression, distant metastasis, short disease-free survival, and short overall survival. However, it does not correlate with the tumor size [4,139,140].

### 9.4. SHH in Triple-Negative Breast Cancer (TNBC)

Canonical SHH signaling plays a role in triple-negative breast cancer (TNBC) [4]. The SHH signaling pathway is a regulator of angiogenesis in TNBC [138]. Mauro et al. identified that angiogenesis of TNBC is regulated by the SHH pathway [138]. In TNBC, some researchers found a correlation between SMO expression and histological grade or tumor stage. Riaz et al. found that expression of SMO corresponds with early onset and subtype of TNBC. Canonical SHH signaling enhances tumor angiogenesis via mechanisms including metalloproteases, CYR61, and VEGF receptor 2 (VEGFR2), resulting in TNBC growth and metastasis [4,131,138,141]. The osteolytic bone metastasis of TNBC is also affected by the SHH pathway [4]. TNBC has a high proportion of basal-like progenitors, which retain primary cilia and GLI1 expression. The ligand-dependent stimulation of canonical SHH pathways affect TNBC [138,142]. In vitro studies reveal that overexpression of SHH enhances cell proliferation, colony formation, migration, and invasion of TNBC [131,143]. Likewise, an in vivo study revealed that such overexpression enhances the growth of orthotopic xenograft and promotes lung metastasis [131].

Some investigators emphasize that GLI1 upregulation mainly affects the maintenance and proliferation of breast CSC. GLI1 activation could upregulate the multidrug-resistant protein-1 (MDR-1), resulting in resistance to doxorubicin, paclitaxel, and cisplatin [4,144]. Recently, Ruiz-Borrego et al. conducted a phase Ib clinical trial study using combined sonidegib (LDE225) (a small molecular oral inhibitor of the SMO/SHH pathway) and docetaxel to treat advanced TNBC patients [145].

### 9.5. SHH in the Microenvironment of Breast Cancer

Aberrant upregulation of SHH affects changes in the tumor microenvironment of breast cancer [4,130], whereas type II non-canonical SHH signaling plays a role in the tumor stroma of breast cancer [4]. The tumor microenvironment/stroma affects tumor development and metastasis [4,5], and the tumor microenvironment/stroma of breast cancer includes endothelial cells, immune cells, adipocytes, and activated fibroblasts (the so-called “cancer-associated fibroblasts” (CAFs)) [6]. CAFs fuel tumor cells via secreting soluble factors [7,8,9,10] to induce metastasis and chemoresistance. This process involves extracellular acidification, inflammation, activation of matrix metalloproteases, and decreased effects of chemotherapeutic drugs [7,11,12]. Tumor microenvironment cells also include tumor-associated macrophages with aberrant genetic and epigenetic changes that may induce a high expression of signaling molecules to enhance the survival of tumor cells [146]. Inhibitors targeting SHH, Notch, CDKs, mTOR, and WNT are promising and are involved in ongoing clinical trials, either in single use or combined use in therapy [146]. Such microenvironment remodeling also activates an antioxidant response in SHH signaling to enhance the CSC in ER-positive BC [147]. A hypoxic microenvironment affects the upregulation of GLI1. In hypoxia, hypoxia-inducible factor 1-alpha (HIF-1α) induces SHH expression in fibroblasts to affect GLI1 induction in a paracrine manner [4,143,144,145,146,147,148].

## 10. SHH in Organogenesis of the Lung and Lung Cancer

### 10.1. Organogenesis of the Lung

SHH is necessary for the growth and differentiation of the trachea and lungs. During lung development, SHH plays an essential role in lung development, specifically for lung specification, primary bud formation, and branching morphogenesis. Mutations in SHH or associated signaling components result in foregut defects in humans [99]. Abnormal secretion of SHH causes severe foregut defects and lung hypoplasia. Pulmonary morphogenesis depends deeply on SHH activation and molecular interactions with other signaling pathways [149]. SHH signaling pathway molecules are required for embryonic lung development [150]. Hypoplastic lungs were found in *SHH*, *GLI1*, *GLI2*, or *GLI3* knockout. PTCH knockout is lethal before lung development begins.

### 10.2. Suppressing the SHH/SMO Pathway to Inhibit Lung Cancer

SHH expression is negatively correlated with tumor differentiation in lung cancer [151]. Patients with higher SHH expression could have a poorer prognosis and worse overall survival [152]. Therefore, SHH could be a prognostic marker. Szczepny et al. reported that an autocrine, ligand-dependent model of the SHH signaling pathway contributes to the pathogenesis of small cell lung cancer [153]. They also found a novel role of non-canonical SHH signaling in producing chromosomal instability [153]. Sun et al. reported that hyperactivated SMO could facilitate the proliferation of non-small cell lung cancer cells [154], and found that HECT and RLD domain containing E3 ubiquitin ligase 4 (HERC4) is inhibited after destabilizing oncoprotein SMO [154].

## 11. Targeting SHH/SMO/GLI Signaling Pathway for Cancer Stem Cells

Cancer stem cells (CSC), a subpopulation of cancer cells with self-sustaining characteristics, play an essential role in tumorigenesis, cancer progression, metastasis, recurrence, and drug resistance [155,156]. The SHH signaling pathway activates in cancer stem cells of several neoplasms such as glioblastoma, as well as cancers of the colon, liver, breast, pancreas, and blood neoplasms (chronic myeloid leukemia and multiple myeloma) [14]. The pathway not only triggers tumorigenesis with uncontrolled cell growth, but also promotes cell migration, mitosis, and can sustain cancer cell survival [14]. Moreover, self-renewal of CSC regulated by the SHH/SMO/GLI signaling pathway has been observed [156].

PTCH1-dependent and SMO-independent (type I non-canonical Hedgehog signaling) paths are both necessary for the survival of CSC [111].

Regan et al. proposed that PTCH1-dependent (non-canonical SHH signaling) positively affects WNT to maintain CSCs with an undifferentiated state [111]. PTCH1 is a dependence receptor that can induce apoptosis even when SHH ligand is absent [157]. However, canonical SMO-dependent SHH signaling, as mediated by GLI1 nuclear localization, downregulates WNT signaling and tumor cell differentiation. Targeting non-canonical SHH signaling to induce CSC differentiation may provide a strategy to eliminate the therapy-resistant CSCs. SHH is therefore proposed as a target in the treatment of SHH-dependent pancreatic cancer and breast cancers [158,159].

SHH/SMO/GLI affecting epithelial–mesenchymal transition allows the transformation of polarized epithelial cells into motile mesenchymal cells, enhancing invasive growth and metastasis [111,155]. Some investigators found that drug transport pump expression in cancer stem cells enabling cytotoxic drug resistance were upregulated by SHH signaling [156]. This is important in the combined use of SHH/SMO/GLI signaling inhibitors and chemotherapy, radiation therapy, or immunotherapy to target CSCs. SMO receptor antagonists may also be able to inhibit this process [14]. Using pharmacological inhibitors that target the SHH/SMO/GLI pathway to inhibit CSC is therefore a promising strategy [155].

## 12. Concluding Remarks

SHH plays an important role in organogenesis, cancer, and the cancer microenvironment of some organs. Combined use of SHH signaling inhibitors and chemotherapy/radiation therapy/immunotherapy could be key in targeting cancer stem cells. Better understanding of these mechanisms could help us better target the SHH pathway against cancer.

## Abbreviations

SHH	Sonic Hedgehog
CSC	Cancer stem cells
GLI	Glioma-associated oncogene homolog
WNT	Wingless-activated
CAFs	Cancer-associated fibroblasts
SMO	Smoothened
GPCR	G protein-coupled receptor
SUFU	Suppressor of fused homolog
HH	Hedgehog
PKA	Protein kinase A
GTPase	Guanosine triphosphatase
PI3K	Phosphoinositide 3-kinase
mTOR	mammalian target of rapamycin
SBE7	Shh brain enhancer 7
VZ	Ventricular zone
TGF1β	Transforming growth factor 1 beta
YAP1	Hippo/Yes-associated protein
HBx	HBV gene product HBx protein
CDK1	cyclin-dependent kinase 1
HCC	Hepatocellular carcinoma
EMT	epithelial-mesenchymal transition
FAK	focal adhesion kinase
TAMs	Tumor-associated macrophages
PDGF	Platelet-derived growth factor
MMP-2	Matrix metalloproteinase-2
HGF	hepatocyte growth factor
VEGFR	Vascular endothelial growth factor receptor
TGF-α	transforming growth factor-alpha
MDB5	2-chloro-N ^1^-[4-chloro-3-(2-pyridinyl)phenyl]-N ^4^,N ^4^-bis(2-pyridinylmethyl)-1,4-benzenedicarboxamide
HP	Helicobacter pylori
slfn4	Schlafen 4
BMPs	bone morphogenic proteins
BrdU	5-Bromo-2′-Deoxyuridine
Bcl-2	B-cell lymphoma 2
c-Src	cellular Src kinase
ERα	Estrogen receptor alpha
ERK	Extracellular signal-regulated kinase
DICS	ductal carcinoma in situ
IBC	invasive breast cancer
CA	carbonic anhydrase
CYR61	Cysteine-rich angiogenic induced 61
ER	estrogen receptor
BC	breast cancer
TNBC	triple negative breast cancer
MDR-1	multidrug resistant protein-1
HIF-1α	Hypoxia-inducible factor 1-alpha
HERC4	HECT and RLD domain containing E3 ubiquitin ligase 4
